# Effect of bilateral low serratus anterior plane block on quality of recovery after trans-subxiphoid robotic thymectomy: Results of a randomized placebo-controlled trial

**DOI:** 10.7150/ijms.91797

**Published:** 2024-05-13

**Authors:** Yu Fu, Huimin Fu, Wei Wei, Huqing Liu, Zongmei Wen, Xin Lv, Yugang Lu

**Affiliations:** 1 Department of Anesthesiology, Shanghai Pulmonary Hospital, School of Medicine, Tongji University, Shanghai, China.; 2 Department of Emergency, Xinhua Hospital Affiliated to Shanghai Jiao Tong University School of Medicine, Shanghai, China.; 3 Department of Ultrasound, 411 hospital, Shanghai University, Shanghai, China.

**Keywords:** Anesthesia, Local, Robot-Assisted Surgery, Thymectomy, Pain Measurement, Quality of Life

## Abstract

**Purpose:** This study aimed to investigate the impact of ultrasound-guided, bilateral, low level (T8-T9) deep serratus anterior plane (DSAP) blocks on postoperative recovery quality and postoperative analgesia in patients undergoing trans-subxiphoid robotic thymectomy (TRT).

**Methods:** 39 patients undergoing TRT were randomized to receive either low DSAP block under general anesthesia (Group S) or the sham block (Group C) on each side. The primary outcome was the QoR-40 score at postoperative day (POD) 1. Secondary outcomes included numeric rating scale (NRS) scores over time, postoperative 48 hours opioid consumption, QoR-40 scores at POD 2, 30, and 90.

**Results:** The QoR-40 scores on POD1-2 were higher in Group S than in Group C [179.1 (4.9) vs 167.7 (2.8), P < 0.01; 187.7 (4.6) vs 178.1 (3), P < 0.01, respectively]. Pain scores were significantly lower in Group S, both during resting and motion at postoperative 6h, 12h, and 24h (P < 0.05 for each). The total amount of sufentanil consumed in the first 48 h was lower in Group S than in Group C [61.4 (4.9) vs 78.9 (4.6), P < 0.001].

**Conclusion:** The bilateral low DSAP blocks enhanced the QoR-40 for 2 days postoperatively, relieved postsurgical pain, and reduced opioid consumption during the early postoperative period in patients undergoing TRT.

## Introduction

Currently, minimally invasive surgical approaches have replaced median sternotomy for the treatment of anterior mediastinal tumors and myasthenia gravis, which have many advantages such as minor trauma, less surgical complications and better postoperative recovery.^1^-^3^ Among the minimally invasive techniques, the robotic subxiphoid approach is a widely used and promising technique.^4^-^6^ The advantages of this technique combining the subxiphoid approach with the da Vinci robotic system are as follows: (Ⅰ) the visual field, comparable to that achievable via sternotomy, can be obtained through the camera scope inserted from the midline camera port; (Ⅱ) the soft tissue in the subxiphoid region, unlike rigid rib structure, which makes it easier to transfer the entire specimen through the surgical incision; (Ⅲ) the cosmetic outcome is superior; and (Ⅳ) the surgical robot provides a magnified three-dimensional and high-definition vision, as well as offers a markedly improved ergonomics including articulating endo-wristed instruments and filtration of physiologic hand tremors.

Initially, Suda *et al.* developed the trans-subxiphoid robotic thymectomy (TRT) in 2015.^4^ Lu *et al.* subsequently developed a new approach via subxiphoid and subcostal arch facilitating the thoracoscopic thymectomy and extended thymectomy.^7^ Now, many treatment centers increasingly perform TRT via subxiphoid and subcostal arch approach.^1^,^8^ In this approach, a 3-cm incision is made below the xiphoid as camera port and two additional trocars are inserted bilaterally below the costal margin. In spite of trauma and postoperative pain in TRT may be minimal due to intercostal spaces are not traversed,^9^ incisions of subxiphoid and subcostal arch can still bring painful feeling after surgery. Inadequately controlled pain after thoracic surgery negatively affects the risk of postoperative complications (such as respiratory and cardiac complications), quality of recovery, length of stay, and the risk of chronic post-surgical pain. 10-12 Therefore, it is important to optimize perioperative pain management to accommodate the transition from sternotomy to minimally invasive surgery (MIS).

Epidural analgesia, as a traditional pain management standard for patients undergoing thoracic and abdominal surgeries, has been questioned in MIS, due to the potential severe complications and strict requirements on coagulation function.^13^ Blanco *et al.* firstly developed serratus anterior plane (SAP) block in 2013, 14 which can block the thoracic intercostal nerves in the lateral part of the thorax to provide an excellent analgesic effect. The SAP block has gained popularity as it is a reliable, efficient and easy to perform analgesic technology with a low risk of adverse effects.^15^,^16^ Described by Blanco and his colleagues,^14^ there are two methods of SAP block: superficial serratus anterior plane (SSAP) block (injection in the plane superficial to the serratus anterior muscles), and deep serratus anterior plane (DSAP) block (injection in the plane between the posterior surface of the serratus anterior muscle and the periosteum of the rib). Practically, the DSAP block to separate the serratus anterior muscle from the rib is easier than the SSAP block to separate the serratus anterior from the external intercostal muscle.

The level of T6-L1 is responsible for the innervation of the abdominal wall, which means that SAP block also provides analgesia for upper abdominal wall incisions by blocking these sensory dermatomes in the thoracic region. Recent studies have demonstrated the good analgesic efficacy of low SAP block in laparoscopic cholecystectomy and hepatectomy. 17,18 However, the efficacy of bilateral low SAP blocks analgesia in the patients undergoing TRT remains unknown. Whether a relationship exists between bilateral low SAP blocks and postoperative recovery quality is still unclear.

Given these knowledge gaps, we designed a randomised controlled trial to assess the impact of bilateral low DSAP blocks on the quality of recovery (QoR) after TRT, and to evaluate the analgesic efficacy of bilateral low DSAP blocks for patients undergoing TRT.

## Material and methods

### Ethics approval and registration

The Clinical Research Ethics Committee of Shanghai Pulmonary Hospital approved this study (approval number K21-372Y) and all subjects who took part in this trial gave written consent. The study was registered at the Chinese Clinical Trial Registry before patient enrollment (ChiCTR2200055315, Principal investigator: Yu Fu, Date of registration: 1 January 2022). Besides, the study protocol was prospectively published in the Journal of Pain Research on 6 April 2022 (DOI: 10.2147/JPR.S359638).^19^ The study was conducted following the Consolidated Standards of Reporting Trials (CONSORT) guidelines.^20^

### Participants

This study was conducted in Shanghai Pulmonary Hospital, School of Medicine, Tongji University between January 2022 and March 2023. To be enrolled, patients had to meet all the following criteria: (1) age range 18-80 yr, (2) ASA grading between I and III, and (3) received elective TRT and signed informed consent for the follow-up study. Exclusion criteria included (1) contraindication to bilateral low SAPB, (2) history of opioid as well as opiate abuse, (3) abnormal coagulation function, (4) preexisting skin infection or eczema at the injection site, (5) preexisting chronic pain, or (6) known psychiatric and neurologic disorders that don't include stable myasthenia gravis (refers to no symptoms or functional limitations from myasthenia gravis but some weakness on examination of some muscles). We invited all eligible patients to participate in this study, and those who enrolled provided written informed consent one day before surgery.

### Randomization and Blinding

A research investigator (H.Fu) who was blinded to study design prepared random numbers using SPSS 26.0 (IBM, Chicago, IL, USA). Participants were randomly assigned to 2 groups at a 1:1 ratio via random numbers. Group allocation concealment was performed using sealed envelopes. On the morning of surgery, the anaesthesiologist (Y.Fu) prepared a 40-mL syringe labelled “research solution” consisting of 0.375% ropivacaine or normal saline according to the group allocation. Before surgery, another anaesthesiologist (Y. Lu) performed the ultrasound-guided bilateral low DSAP blocks using “research solution” for each participant. Blocks were performed under general anesthesia, which meant that the patients were not aware of their group allocation, and resulting in the absence of sensory evaluation of the block. Healthcare workers (H.Liu and Z.Wen) who were involved in data collection were also blinded to the group allocation and were not granted access to the randomization until the completion of the data analysis. Thus, this study was conducted with a double-blind design.

### Standard anaesthesia and analgesia protocol

Standard perioperative care was given to all patients. After attaching the standard monitoring of electrocardiogram (ECG), pulse oximetry (SpO2), and non-invasive blood pressure (BP), the right internal jugular vein cannula was placed and Ringer's solution infusion was started in the operating room. Induction of anesthesia was done by standard doses of midazolam, propofol, sufentanil, and rocuronium bromide. This was followed by double-lumen endotracheal intubation to achieve lung isolation. Anaesthesia was maintained with total intravenous anesthesia, with the objective of attaining a bispectral index value ranging between 40 and 60. The administration of remifentanil was initiated at a dose of 0.1 μg/kg/min, and then adjusted according to the hemodynamic parameters, with a maximum of 2 μg/kg/min. Specifically, the anesthetic, liquid volume and rate of infusion were adjusted in accordance with hemodynamic monitoring conditions to keep the systolic BP and HR within 20% of the baseline. An electronic anaesthesiology recording system (Medicalsystem Co., Ltd., Nanjing, China) was used to document vital signs. After the surgical procedure, the neuromuscular blockade was reversible by utilizing a combination of neostigmine and atropine to meet the criteria for extubation. Patients were transferred to the post-anesthesia care unit (PACU) for one hour after extubation and then to the surgical ward. As a component of multimodal analgesia, all patients have been administered 50 mg flurbiprofen intravenously 30 minutes prior to the surgical incision and immediately upon their arrival in the PACU.

Patients received intravenous opioid patient-controlled analgesia during the first 48 hours after surgery. The PCIA pump was set up with sufentanil 100 μg in 0.9% sodium chloride 100 mL, at the rate of 2 mL/hour, with a single bolus dose of 2 μg, and a lockout period of 15 minutes. Intravenous flurbiprofen 50 mg was administered as a rescue analgesic to patients if the NRS ≥ 4, and it could be repeated if necessary (maximum, not exceeding 200 mg/day).

### Ultrasound-guided bilateral low DSAP blocks

The bilateral low DSAP blocks were performed by the experienced anesthesiologist (Y.Lu) under ultrasound guidance after anesthesia induction. The patient was supine and the ultrasound probe (8-12 MHz) was placed on the sagittal plane of the midclavicular line of the chest. The probe was moved downward to count the ribs until the level of 8-9th inter-costal space in the mid-axillary line. The serratus anterior muscle and intercostal muscle were recognized using ultrasound. Then, the “research solution” was injected into the plane between the posterior surface of the serratus anterior muscle and the periosteum of the rib using an in-plane approach with a 22-gauge needle (22G, 80 mm, UniPlex®, NanoLine®, Germany). The procedure was performed bilaterally for the patients. Thus, a total amount of 40 ml of 0.375% ropivacaine or normal saline was divided into two equal volumes and injected at each side. The injection sites were gently pressed to accelerate the diffusion.

### The Procedure of TRT

All the operations were executed by a fixed surgical team employing the da Vinci surgical system (Surgical Intuitive, Mountain View, CA, USA) with three arms. The lower edge of the xiphoid process, which served as the camera port, was cut with an approximately 30-mm longitudinal incision. Two 8-mm subcostal incisions, where the da Vinci arms were mounted, were made bilaterally on the midclavicular line. After resection, the thymus was placed in a bag dedicated to mediastinal tumors and removed through the subxiphoid incision.

### Outcome measures

The primary outcome was the global Quality of Recovery-40 (QoR-40) score on postoperative days (POD) 1. The QoR-40 scale is a reliable measure of a patient's quality of recovery after surgery, which scores ranging from 40 to 200.^21^ Secondary outcomes included: (1) NRS scores at rest and with movement at 6 h, 12 h, 24 h, 48 h, 30d, and 90d postoperatively; (2) cumulative opioid consumption within 48 h postoperatively; (3) PCIA pump use within 48 h postoperatively; (4) use of rescue analgesics within 48 h postoperatively; (5) QoR-40 scores at POD 2, 30, and 90; and (6) incidence of adverse events such as block complications, nausea and vomiting, sedation, pruritus, and surgical complications.

### Sample size and statistical analyses

The established minimum clinical improvement in recovery quality after surgery is 10-points in the global QoR-40 score. 22,23 Based on our pilot study in a population of patients undergoing TRT, which reported that the global QoR-40 score at the 24th hour postoperatively was 168 (10), the sample size was calculated. Assuming α = 0.05 and β = 0.20 for a 10-point difference in global QoR score between groups, the calculated sample size was 17 subjects per group. In consideration of possible dropouts, a total of 40 patients (20 patients in each group) were recruited. The calculation of sample size was made using the software G*Power (version 3.1).

Statistical Product and Service Solutions (SPSS) version 26.0 (IBM SPSS Inc., Chicago, IL, USA) was used for all statistical analyses. The normal distribution of the variables was assessed using the Shapiro-Wilk test, and if it was satisfied, the data were presented as mean (standard deviation, SD), and analyzed with an independent sample t-test (Student's t-test) between groups. Otherwise, the data were presented as median (interquartile range, IQR), and analyzed with the Mann-Whitney U-test between groups. Categorical variables were presented as frequency (percentage), and compared using the chi-square test or Fisher's exact test. Analysis of variance (ANOVA) was used for multiple testing. Post hoc multiple comparisons were conducted using the Bonferroni method when significant interactions are detected using ANOVA. Two-tailed P-values of less than 0.05 was accepted as statistically significant.

## Results

**Figure 1:**
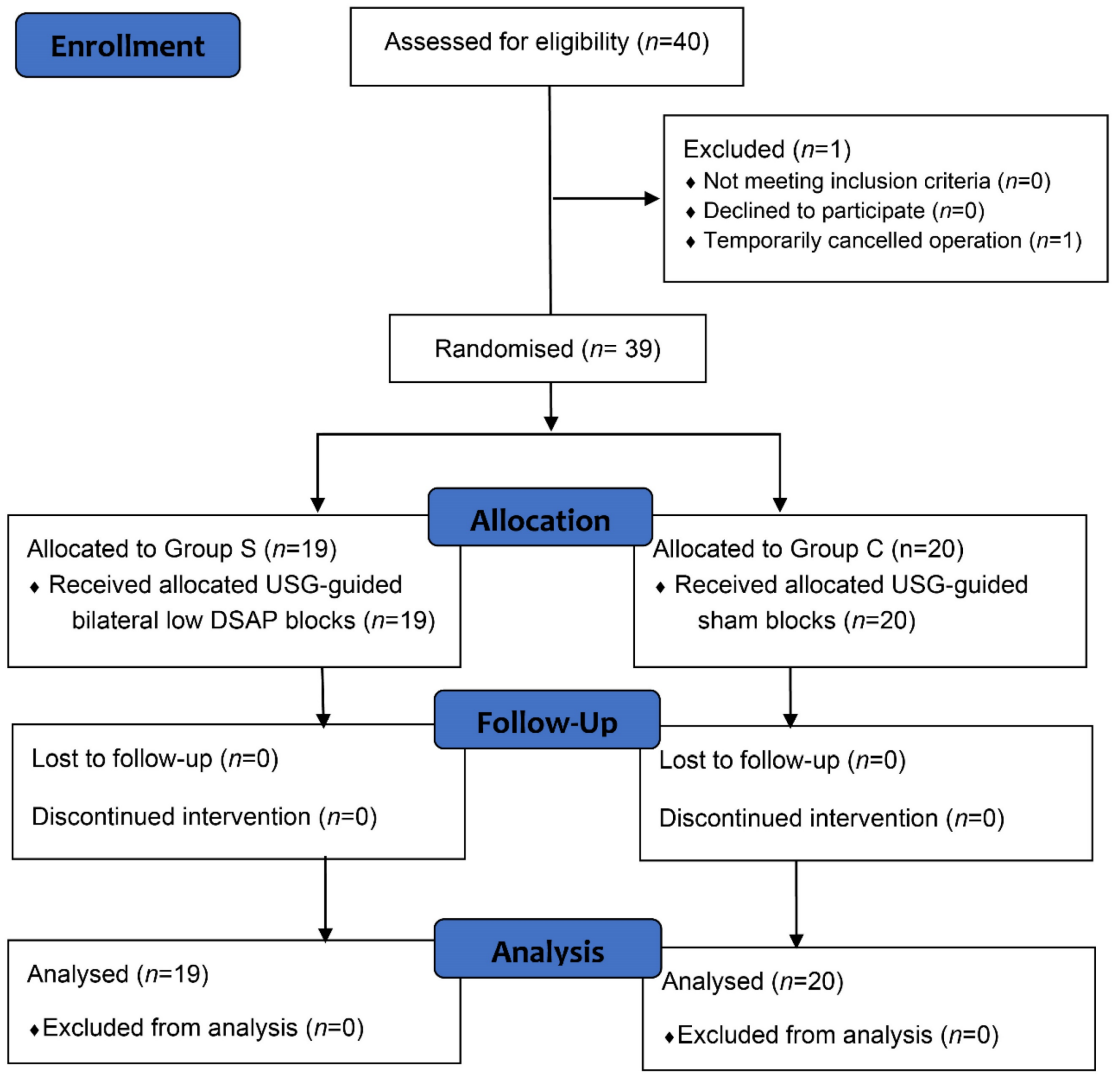
**Consolidated Standards of Reporting Trials flow study diagram describing patient progress through the study. Abbreviations:** DSAP, deep serratus anterior plane; USG, ultrasonography.

In this study, 40 patients were initially assessed for eligibility. However, one patient was excluded because temporarily cancelled operation. The remaining 39 cases were equally and randomly allocated to the bilateral low DSAP blocks or placebo control groups (Group S, n=19; Group C, n=20). No subjects were lost to follow-up (**Figure 1**). Both groups had similar patient characteristics and duration of surgery (**Table 1**).

### The Quality of Recovery-40

**Figure 2:**
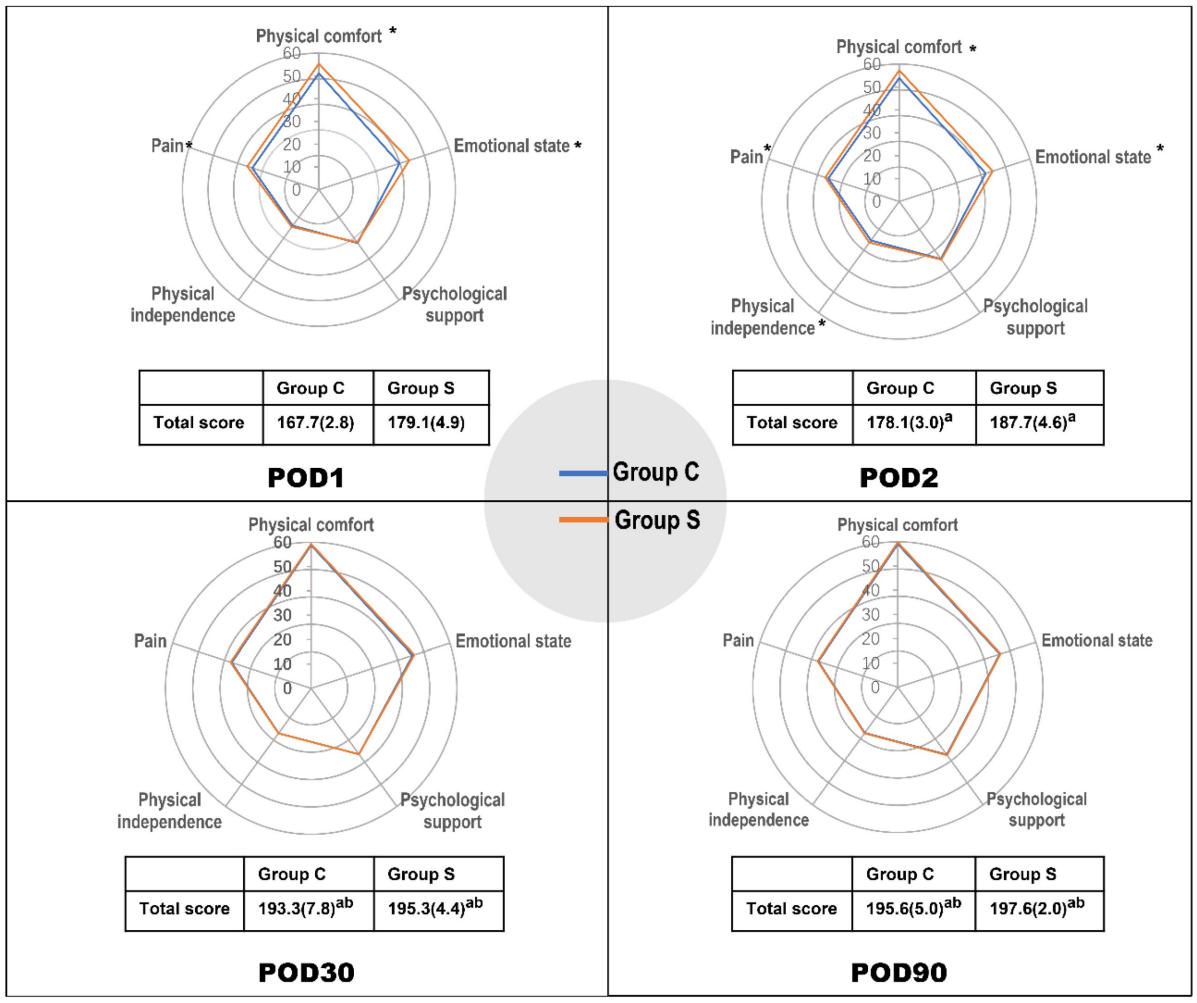
**Postoperative QoR-40 scores at POD1, 2, 30, and 90.** The data are represented as mean (SD). Data collected at multiple points in time were analyzed using repeated-measures ANOVA. *P < 0.05 compared with Group C. ^a^P < 0.05 compared with POD1. ^b^P < 0.05 compared with POD2. **Abbreviations:** POD, postoperative days.

The postoperative global and dimensional QoR-40 scores are showed in **Table 2** and **Figure 2**. This measurement was repeated at POD1, 2, 30, and 90. The repeated-measures ANOVA showed that there was interaction between time and group (P < 0.001), so Bonferroni correction was applied at each time point. The total QoR-40 scores of both groups at POD2, 30, and 90 were higher than those at POD1 (P < 0.001). Similarly, the total QoR-40 scores of both groups at POD30 and 90 were higher than those at POD2 (P < 0.001). However, there were no significant differences in the total QoR-40 scores of both groups between POD30 and 90 (P > 0.05; **Figure 2**). The global QoR-40 scores on both POD1 and 2 were significantly different between the groups (POD1: mean difference -11.5[95% confidence interval (CI): -14.1 to -8.8], P < 0.01, and POD2: -9.7[95% CI: -12.2 to -7.2], P < 0.01). Among the five dimensions, the scores of physical comfort, emotional status, and pain dimension were higher in Group S at POD1 and 2 than in Group C (P < 0.01 for each), moreover, the physical independence dimension score was significantly higher in Group S at POD2 than in Group C (P < 0.05; **Table 2**).

### Numeric Rating Scale

**Figure 3:**
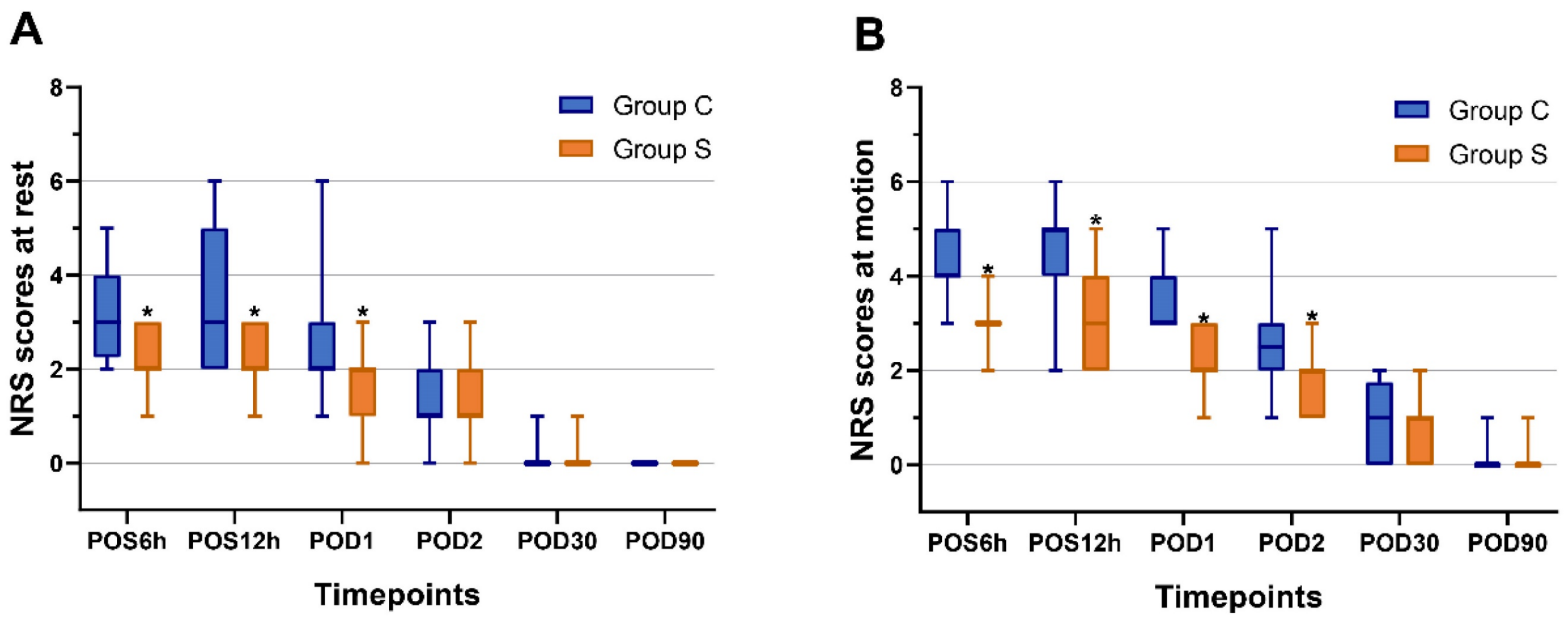
**Postoperative numerical rating scores (A) at rest and (B) at motion.** Boxes present interquartile ranges while whiskers show 1 to 99 percentiles. The Mann-Whitney U test was used in the comparison between groups. *P < 0.05 compared with Group C. **Abbreviations:** NRS, numeric rating score; POS, postoperative; POD, postoperative days.

The ultrasound-guided bilateral low DSAP blocks was performed successfully in all subjects. No complications were attributable to regional anaesthesia were observed. The repeated-measures ANOVA showed that time effect, group effect, and time-group interaction were statistically significant regarding pain scores at rest and motion (P < 0.01 for each). The NRS scores were statistically significantly lower in Group S compared to Group C, both during resting and motion at POS6h, POS12h, and POD1 (P < 0.05 for each). Furthermore, the NRS scores remained lower in Group S compared to Group C during motion at POD2 (P < 0.05; **Figure 3**).

### Other outcomes

As detailed in **Table 3**, the number of PCIA button presses within 48h was significantly lower in the Group S compared to Group C (P < 0.001). Similarly, the cumulative sufentanil and rescue analgesic consumption within 48h was significantly lower in the Group S compared to Group C (P < 0.001 and P < 0.05, respectively). No significant differences in postoperative complication rates were found between Group S or Group C. The postoperative nausea or vomiting (PONV) was the most common postoperative complications observed (**Table 3**).

## Discussion

The present study is the first randomized controlled trial comparing the ultrasound-guided bilateral low DSAP blocks versus the sham block in TRT. Our results indicated that patients who received the ultrasound-guided bilateral low SAPB had higher global QoR-40 scores at POD1 and 2, which showed that they had a better quality of recovery after TRT. Furthermore, we demonstrated that patients exposed to bilateral low DSAP blocks prior to surgery felt less pain in the early postoperative period and decreased cumulative opioid consumption following surgery.

Robotic surgery, a new surgical frontier, offers a minimally invasive platform to treat a wide variety of complex thoracic diseases. 24,25 Robot-assisted thymectomy via the subxiphoid approach has increasingly attracted professional and public interest. 1,26 As mentioned above, these involved incisions of TRT are limited to the upper abdominal wall, ranging from the T6 to T10 intercostal nerves.^19^ With advancements in medical devices and techniques enabling the surgical resolution of intricate intrathoracic pathology in patients, anesthesiologists should devote greater attention to the improvement of efficient, safe analgesic management and patient-oriented quality of recovery. However, scientific literature about pain management for patients undergoing TRT remains limited.

Despite epidural analgesia (EA) having a long history of use in thoracic and abdominal surgeries, which keeps declining in popularity currently owing to a reportedly high failure rate and the potential multitude of complications.^27^,^28^ Several ultrasound-guided regional techniques may be preferred for effective safe alternatives to thoracic EA, including interfascial plane blocks. SAP block is regarded as a promising interfascial plane block owing to its ease of execution and capacity to completely cover the surgical area affected by thoracoscopic surgery.^15^,^29^ Additionally, it has been shown that the utilization of low SAP block can provide a good analgesic effect and reduce opioid consumption in liver surgery and laparoscopic cholecystectomy.^17^,^18^ Thus, we speculate that the ultrasound-guided bilateral low DSAP blocks can improve the quality of recovery and provide an efficient analgesic effect for patients undergoing TRT.

Modern recovery after surgery involves a process that ultimately results in the patient returning to a relative state of normality, independence, optimal well-being, and self-efficacy. Although lower pain scores hold significant importance after surgery, they won't be seen as a better recovery experience if they come with other negative side effects. Hence, we focused on how well the patient recovered, which is assessed by QoR-40 scores. The QoR-40 questionnaire is acknowledged as the most widely used scale for assessing patients' quality of recovery. 21,30,31 This study showed that there was a statistically significant difference in global QoR40 scores of 11.5 points at POD 1, this succeeded in reaching our predefined primary outcome of a 10-point difference. Besides, there remained a statistically significant difference in global QoR40 scores of 9.7 points at POD 2. Our results pointed out that the bilateral low DSAP blocks brought about a significantly better early postoperative recovery of patients after TRT.

A potent postoperative analgesia is important for recovering from surgery. Although published articles concluded that SAP block is now wildly used in postoperative analgesia treatment of the chest wall including breast cancer surgery,^32^ thoracoscopic surgery,^33^ and thoracotomy,^34^ there is still limited clinical data on SAP block used in upper abdominal surgery. Tao *et al.*^17^ reported that the unilateral SAP block (T6-T7) offered excellent analgesia and reduced perioperative opioid consumption for open partial hepatectomy. And Wu *et al.*^18^ reported that the unilateral low SAP block (T8-T9) reduced the postoperative pain score for patients undergoing laparoscopic cholecystectomy. Given that surgical incisions of TRT are limited to the upper abdominal wall, the bilateral low level (T8-T9) SAP blocks may be utilized for pain relief following the subxiphoid incision and bilateral subcostal incisions. In the present study, we found that the bilateral low DSAP blocks did provide superior postoperative early analgesia to patients undergoing TRT. However, there were no disparities observed in the NRS score between the two groups on POD30 and 90. This may have been because our sample size is insufficient to compare the incidence of chronic postsurgical pain.

This study also yields significant results regarding the opioid-reducing of preoperative bilateral low DSAP blocks. In this study, we observed that the bilateral low DSAP blocks offered a reduction of the cumulative sufentanil consumption within postoperative 48h compared to the control. There are several negative effects of opioids, including respiratory depression, postoperative nausea/vomiting, and the possibility of developing an addiction. Therefore, it is necessary to minimize the usage of opioids to the public. This is in line with the ongoing campaign for the active participation of medical professionals in reducing the narcotic abuse/overdose epidemic. 35,36

There were several limitations in this study. First, the blocks were performed under general anaesthesia. Thus, the distribution of sensory blockade was not assessed, which increased the possibility that some blocks weren't entirely effective. However, this procedure that performs ultrasound-guided peripheral nerve blocks following the induction of general anaesthesia is in line with the established clinical practice. Moreover, while performing the blocks, we observed a hypoechoic ellipsoid with a well-defined margin beneath the serratus anterior muscle on ultrasonic view in each patient. Hence, our findings ought to hold significance for widespread application. Second, the present study employed the Chinese-written version of the QoR-40 questionnaire, which may have language and cultural influences on the results of this study. However, this version has been formally validated with good reliability, validity, and responsiveness.^37^ Third, as the blocks was administered preoperatively, it is indeed possible that the managing and blinded anesthesiologist may have become unblinded due to the need for opioids intraoperatively. Actually, in our study, the intraoperative anesthesiologist was allowed to adjust the dose of remifentanil according to hemodynamic parameters during intraoperative procedure. Besides, the liquid volume and rate of infusion were adjusted in accordance with hemodynamic monitoring conditions to keep the systolic BP and HR within 20% of the baseline. If a patient was suspected to have severe hemodynamic change that could not be corrected, we would initiate unblinding. We carefully monitored the anesthetic and intraoperative management to mitigate any potential bias resulting from unblinding. Until the end of this study, no unblinding events occurred. Despite this limitation, we believe that the robust design and statistical analysis of our study provide meaningful insights into the efficacy and safety of bilateral low DSAP blocks. Finally, many clinically relevant issues require further investigation, such as the difference between a superficial or deep anterior serratus plane block in patients who have undergone TRT; and the impact of employing diverse volumes and concentrations of local anaesthetic solutions and additives.

In conclusion, this single-center, prospective double-blind, randomised controlled trial among TRT patients has shown that patients who received the bilateral low DSAP blocks enhanced the quality of recovery, relieved postsurgical pain, and reduced opioid consumption during the early postoperative period. Our findings will encourage the use of low DSAP blocks in patients undergoing TRT, serving as an effective method for alleviating postoperative pain and fostering recovery.

## Figures and Tables

**Table 1:** Comparison of baseline characteristics and surgery duration between the two groups.

Variable	Group C (n=20)	Group S (n=19)	*P*-value
Age, yr	52.9 (10.1)	54 (12.4)	0.752
Gender, n (%)			0.621
Female	10 (50)	11 (57.9)	
Male	10 (50)	8 (42.1)	
Height, cm	164.9 (7.1)	164.8 (8.0)	0.981
Weight, kg	64.9 (9.4)	67.4 (6.9)	0.357
Body mass index, kg/m2	23.8 (2.2)	24.8 (2.0)	0.140
ASA physical status, n (%)			0.242
Ⅰ	10 (50)	6 (31.6)	
Ⅱ	10 (50)	13 (68.4)	
Duration of surgery, min	94.5 (31.6)	85.1 (28.4)	0.337

The data are represented as n (%) or mean (SD). Abbreviations: ASA, American Society of Anesthesiologists.

**Table 2:** Postoperative QoR-40 scores at POD1 and POD2.

QoR-40	Group C (n=20)	Group S (n=19)	*Between-group P*-value
**POD1**			
Total score	167.7 (2.8)	179.1 (4.9)	<0.01
Physical comfort	51.2 (3.0)	55.4 (1.8)	<0.01
Emotional state	37.2 (3.3)	41.8 (1.6)	<0.01
Psychological support	28 (27-31.5)	28 (28-30)	0.596
Physical independence	19 (17.5-21)	20 (18-21.5)	0.249
Pain	31 (30-32)	33 (32-34)	<0.01
**POD2**			
Total score	178.1 (3.0)	187.7 (4.6)	<0.01
Physical comfort	53.9 (2.1)	57.3 (1.8)	<0.01
Emotional state	40 (37.5-41)	43 (42-44)	<0.01
Psychological support	31 (2.4)	31.3 (2.7)	0.703
Physical independence	21 (2)	22.2 (1.3)	0.024
Pain	33 (32-34)	34 (34-34)	<0.01

The data are represented as mean (SD) or median (IQR). The mean (SD) data were compared with the independent t test. The median (IQR) data were compared using the Mann-Whitney U test. Abbreviations: QoR-40, Quality of Recovery-40; POD, postoperative days.

**Table 3:** Comparison of other perioperative outcomes between groups.

Outcomes	Group C (n=20)	Group S (n=19)	*P*-value
Times PCIA used within 48h	3 (3-4)	1 (1-2)	<0.001
Cumulative sufentanil consumption within 48h, μg	78.9 (4.6)	61.4 (4.9)	<0.001
Rescue analgesic (flurbiprofen) consumption within 48h, mg	50 (50-100)	50 (0-50)	0.01
Postoperative complications, n (%)			
PONV	7 (35)	4 (21.1)	0.48
Pruritus	1 (5)	1 (5.3)	1
Dizziness	3 (15)	1 (5.3)	0.61
Pneumonia	1 (5)	0	1
Arrhythmia	0	1 (5.3)	0.49
Postoperative 30-day readmission	0	0	NS

The data are represented as n (%), mean (SD) or median (IQR). Abbreviations: PONV, postoperative nausea or vomiting; PCIA, patient-controlled intravenous analgesia.
